# Validity and Reliability of the Self‐Care of Chronic Illness Inventory and Self‐Care Self‐Efficacy Scale in Patients Living With Cancer

**DOI:** 10.1111/jan.16823

**Published:** 2025-02-19

**Authors:** Marco Di Nitto, Angela Durante, Francesco Torino, Tatiana Bolgeo, Vincenzo Damico, Greta Ghizzardi, Sipontina Rita Zerulo, Rosaria Alvaro, Ercole Vellone, Valentina Biagioli

**Affiliations:** ^1^ Department of Health Sciences University of Genoa Genoa Italy; ^2^ Sant'Anna School of Advanced Studies Health Science Interdisciplinary Center Pisa Italy; ^3^ Fondazione Toscana “Gabriele Monasterio” Pisa Italy; ^4^ Department of Systems Medicine, Medical Oncology Tor Vergata University of Rome Rome Italy; ^5^ Research Training Innovation Infrastructure–Department of Research and Innovation Azienda Ospedaliero Universitaria SS Antonio e Biagio e Cesare Arrigo Alessandria Italy; ^6^ Hospital–University “Manzoni” of Lecco Lecco Italy; ^7^ School of Nursing, Directorate of Nursing and Allied Health Professions Azienda Socio–Territoriale di Lodi Lodi Italy; ^8^ Azienda Ospedaliera Universitaria Policlinico Riuniti di Foggia Foggia Italy; ^9^ Department of Biomedicine and Prevention Tor Vergata University of Rome Rome Italy; ^10^ Department of Nursing and Obstetrics Wroclaw Medical University Wroclaw Poland; ^11^ Department of Medical and Surgical Sciences—DIMEC University of Bologna Bologna Italy

**Keywords:** adherence, chronic illness, compliance, oncology nursing, psychometrics

## Abstract

**Aim:**

This study aimed to test the psychometric properties of the Self‐Care of Chronic Illness Inventory and the Self‐Care Self‐Efficacy scale in patients with cancer.

**Design:**

A multisite cross‐sectional validation study was conducted.

**Methods:**

Between November 2022 and July 2023, a convenience sample of 318 patients with cancer were enrolled in five Italian inpatient and outpatient facilities. Confirmatory factor analysis was performed on the three scales of the Self‐Care of Chronic Illness Inventory and the Self‐Care Self‐Efficacy scale. Internal consistency was tested using Cronbach's alpha for unidimensional scales and McDonald's Omega for multidimensional scales. Construct validity was assessed with the global health status by Pearson's correlation. The COnsensus‐based Standards for the selection of health Measurement INstruments reporting guidelines were followed for the reporting process.

**Results:**

Three hundred fourteen patients were included (median age: 55.5 years; male: 53.82%). Confirmatory factor analysis showed supportive fit indices for the three Self‐Care of Chronic Illness Inventory scales (CFI: 0.977–1.000; SRMR: 0.004–0.78) and the Self‐Care Self‐Efficacy scale (CFI: 1.000; SRMR: 0.014). All scales demonstrated adequate internal consistency (0.89–0.99) and test–retest reliability (0.85–0.95). Construct validity was confirmed through correlations between Self‐Care Self‐Efficacy, each Self‐Care of Chronic Illness Inventory scale, and global health status.

**Conclusion:**

The Self‐Care of Chronic Illness Inventory and Self‐Care Self‐Efficacy scales demonstrated excellent psychometric qualities and construct validity when administered to patients with cancer. Future research should explore self‐care behaviours across different diseases and cultural contexts.

**Implications For the Profession:**

These tools can help develop targeted educational programs, improving patient outcomes.

**Impact:**

Currently, there is a lack of knowledge regarding self‐care behaviours in patients with cancer. These tools enable healthcare professionals to identify patient needs, design personalised interventions, and monitor their effectiveness over time.

**Patient or Public Contribution:**

No patient or public contribution.

**Summary**

- What is already known?
  ○ Self‐care is essential for managing chronic illnesses, including cancer, as it can influence health outcomes and quality of life.
  ○ Validated tools to measure self‐care behaviours and self‐efficacy are crucial for assessing patients' capacity to manage their condition.
  ○ The Self‐Care of Chronic Illness Inventory and Self‐Care Self‐Efficacy scales have demonstrated reliability in chronic conditions but required validation in cancer populations.
- What this paper adds?
  ○ The validity of Self‐Care of Chronic Illness Inventory and Self‐Care Self‐Efficacy scales in cancer patients is confirmed.
  ○ Self‐Care of Chronic Illness Inventory and Self‐Care Self‐Efficacy demonstrated strong internal consistency and test–retest reliability.
  ○ All the scales confirmed adequate construct validity with the global health status.
- What this paper adds?
  ○ These validated tools can be used in oncology settings to assess patients' self‐care behaviours and self‐efficacy, enabling more personalised care strategies.
  ○ Healthcare providers can use these scales to identify areas where patients need additional education or support to improve self‐care behaviours.
  ○ Incorporating these tools in clinical practice may enhance patient empowerment and lead to better health solutions in cancer care.

## 1 Introduction

Cancer accounted for 19.3 million new cases and 10 million deaths worldwide in 2020 (Ferlay et al. 2021). Cancer prevention is a major public health challenge of the 21st century due to its increasing global burden. In addition to cancer screening, the European Code Against Cancer recommends adopting self‐care behaviours consisting of healthy lifestyle changes in dietary patterns, reducing alcohol consumption, increasing physical activity and maintaining a healthy body weight (Schüz et al. 2015).

Self‐care is a powerful tool that empowers patients to take control of their health. It involves monitoring symptoms related to the illness and treatment and managing the challenges of daily life (Riegel et al. 2012). Patients who practice self‐care feel more responsible and empowered in managing even complex medication regimens, as they retain control over therapy and become less dependent on health professionals.

Self‐care has been extensively studied in patients with chronic conditions, including diabetes mellitus (Ausili et al. 2017), heart failure (Vellone et al. 2020), chronic obstructive pulmonary disease (Clari et al. 2017), and those with colostomy or urostomy (Villa et al. 2018). Its potential in the cancer care community has recently gained attention (Biagioli et al. 2021). Indeed, self‐care can be helpful for patients living with cancer, especially those who are in a chronic phase of their condition, since it includes monitoring symptoms related to the illness and treatment and allows dealing with the challenges of daily life (Riegel et al. 2012).

To harness this potential, it is crucial to have valid and reliable tools to measure self‐care. Hence, a multisite cross‐sectional study was conducted to validate the Self‐Care of Chronic Illness Inventory (SC‐CII) and Self‐Care Self‐Efficacy (SC‐SE) scale, two instruments commonly used in chronic care settings for assessing self‐care and self‐care self‐efficacy, among cancer patients undergoing oral anticancer agents (OAAs).

## 2 Background

According to the Middle‐Range Theory of Self‐Care of Chronic Illness by Riegel et al. (2012), self‐care is a process patients engage in to maintain their physiological and emotional well‐being despite their illness (self‐care maintenance). Self‐care monitoring involves monitoring for any symptoms of their illness and its treatment, while self‐care management means taking necessary action to manage the symptoms and prevent further worsening of the condition (Riegel et al. 2012).

Few studies have been conducted on self‐care in patients living with cancer, and most have been focused on adherence to pharmacological treatment and monitoring side effects (Arthurs et al. 2015). A comprehensive approach to studying self‐care in this population, which considered also lifestyle modification and non‐pharmacological habits to maintain and manage the disease, has received little attention (Howell et al. 2021).

According to a recent qualitative study, patients with cancer may not always be able to recognise symptoms and take appropriate actions to manage their disease (Di Nitto, Sollazzo, Biagioli, Torino, et al. 2022). It is crucial for these patients to correctly recognise and interpret symptoms as it not only plays a significant role in their self‐care process but also in their cancer care (Riegel et al. 2019).

Supporting patients in appropriate self‐care activities can improve their quality of life significantly (Haase et al. 2021). Additionally, self‐care support can help reduce symptom burden (Huang et al. 2023), control the progression of the disease (Caro‐Bautista et al. 2020), and alleviate psychological distress (Yahaya et al. 2022). This, in turn, can reduce the likelihood of hospital admissions (Schrijver et al. 2022) and mortality rates (Basch et al. 2017).

To provide self‐care support to patients with cancer, health professionals need to be able to evaluate the level of patient engagement in each recommended self‐care activity over time (Chan et al. 2023). Moreover, nurses should enhance patients' self‐efficacy, which refers to their confidence in carrying out self‐care actions, as it has been shown to be a significant predictive factor of self‐care and other outcomes (Iovino et al. 2023).

Patient‐reported outcomes, such as self‐care and self‐efficacy, can be obtained using self‐report questionnaires, which help collect data. However, it is important to note that self‐report questionnaires rely on patients' subjective responses, which may not always accurately reflect their actual behaviours or feelings. The Self‐Care of Chronic Illness Index v.2 (SC‐CII) is the most commonly used questionnaire to assess self‐care behaviours that are commonly recommended for various chronic conditions (Arapi et al. 2023; Durán‐Gómez et al. 2023; Riegel et al. 2018). The SC‐CII comprises three scales assessing self‐care maintenance, monitoring, and management. This valid and reliable tool has undergone cross‐cultural validation, indicating that significant comparisons can be made across nations (De Maria et al. 2021). However, the SC‐CII has never been used to assess self‐care behaviours in patients with cancer, so its psychometric properties have not been evaluated in this population. Similarly, the Self‐Care Self‐Efficacy Scale (SE‐SC) is a widely used tool to measure patients' overall confidence in their ability for self‐care maintenance, monitoring, and management (Yu et al. 2021). However, its psychometric robustness in evaluating self‐care self‐efficacy in patients with cancer has not been thoroughly assessed (Fridriksdottir et al. 2023). Validating these instruments for this specific population could provide a standardised and reliable method for assessing self‐care and self‐efficacy in patients with cancer and could potentially improve the quality of care provided. This is especially important in certain clinical settings (e.g. home care) where it is required to care for patients with multiple chronic conditions.

### 2.1 Aim

This study aimed to assess the psychometric properties of the two instruments used to measure self‐care and self‐care self‐efficacy, namely SC‐CII and SE‐SC, in patients with cancer who are receiving OAAs.

## 3 Method

### 3.1 Design

This study is part of a longitudinal study aimed at investigating self‐care behaviours in patients on OAAs (*Article under consideration elsewhere*). The COnsensus‐based Standards for the selection of health Measurement INstruments (COSMIN) reporting guidelines were used to ensure accurate and reliable reporting (Gagnier et al. 2021).

### 3.2 Setting and Sample

A convenience sample of patients with cancer was enrolled from November 2022 to July 2023 across five inpatient and outpatient facilities in Italy (Rome, Foggia, Alessandria, Lecco, and Lodi). The patient's inclusion criteria were: (1) adult patients (≥ 18 years); (2) diagnosis of metastasized or locally advanced solid tumour; (3) space‐temporal orientation; (4) understanding of the Italian language; and (5) active treatment with OAAs (conventional chemotherapy, molecular targeted therapy, or hormone therapy) for at least 3 months. Only patients with metastases or locally advanced cancer were included, as these patients often require long‐term oral anticancer therapy to manage their disease. The medication, unless intolerance occurs, can potentially be used for life. Therefore, self‐care behaviours play a central role in this population and may contribute to prolonged survival. Patients with haematological malignancies were excluded due to their specific and different self‐care behaviours compared to patients with solid tumours. The sample size estimation followed a commonly accepted rule of thumb, requiring a minimum of 200 individuals, which is considered sufficient for an effective Confirmatory Factor Analysis (CFA) (Kline 2023).

### 3.3 Instruments

An ad‐hoc questionnaire was used to collect socio‐demographic information (e.g. age, gender) and clinical characteristics (e.g. type of OAA, tumour site) from the sample.

The SC–CII V. 2 was used to measure self‐care behaviours in chronic conditions (Riegel et al. 2018). This is a 19‐item instrument consisting of three separate scales assessing self‐care maintenance (7 items), self‐care monitoring (5 items), and self‐care management (7 items). Each item is rated on a 5‐point Likert scale ranging from 1 (never) to 5 (always). The score on each scale ranges from 0 and 100, with higher scores indicating better self‐care. Scores ≥ 70 indicate adequate self‐care. The Self‐care management scale, evaluating the extent to which patients adopt specific behaviours to respond to symptoms, can be completed only if patients report symptoms occurring during the last month due to the chronic illness.

The Self‐Care Self‐Efficacy Scale (SE‐SC) is a reliable and valid tool to measure self‐efficacy (Yu et al. 2021). It consists of 10 items and is designed to assess the extent to which patients feel confident in performing self‐care activities (e.g. confidence in following the prescribed treatment plan). Each item uses a 5‐point Likert scale, with responses ranging from 1 (not confident) to 5 (extremely confident). Scores are transformed to a standardised score from 0 to 100, with higher scores indicating greater self‐efficacy.

### 3.4 Data Collection

Trained nurse research assistants identified eligible inpatients and outpatients from various Italian health institutions according to predefined inclusion and exclusion criteria. Participants deemed eligible were provided with detailed information about the study and invited to participate. Data collection began only after the patient signed a consent form, providing their informed consent.

#### 3.4.1 Ethical Considerations

This study adhered to the Good Clinical Practice Standards of the European Union and the Helsinki Declaration. Before starting data collection, this study was approved by the Ethics Committee of the ‘Policlinico Tor Vergata of Rome’ on 20/09/2022 with reference number 188.22. Participation in this study was voluntary. Participants were fully informed regarding the study aims and could withdraw from the study at any time. Participants were enrolled only after signing the informed consent form.

### 3.5 Statistical Analysis

Descriptive statistics were used to describe the sociodemographic and clinical characteristics of the patients who were enrolled in the study. Skewness and kurtosis were calculated to determine the distribution of the items in the SC‐CII and SE‐SC scales. Out of the total sample of 318 patients, four cases were excluded from the analysis as they reported missing responses on all items of the SC‐CII. Since the missing data in SC‐CII items were less than 5%, only complete cases were used for analysis. All analyses were performed using R (version 4.3.1) along with the ‘lavaan’, ‘semPlot’ and ‘semTools’ packages.

### 3.6 Confirmatory Factor Analysis

Confirmatory Factor Analysis (CFA) was conducted to assess the dimensionality of the SC‐CII and SE‐SC. For the SC‐CII a multidimensional model was tested based on the Middle‐Range Theory of Self‐Care of Chronic Illness (Riegel et al. 2012). For SE‐SC, a single‐factor model was tested (Yu et al. 2021). Regarding the SC‐CII, we performed three separate CFA, one for each scale of the SC‐CII (self‐care maintenance, monitoring, and management scale) and one considering all items of the instrument, in line with previous validation studies (De Maria et al. 2021; Riegel et al. 2018). CFA was chosen as the most suitable analysis method as the instruments are theory‐based. Specifically, if a strong correlation among factors emerges, a second‐order model can be tested. First, the multivariate normality was assessed using Mardia's test. CFA was conducted using the unweighted least square (ULS) estimator to account for the non‐normal distribution of the items. This estimator was chosen as it was found to produce more accurate and precise factor loadings than other estimators used for ordinal data (Li 2016). In particular, for the SC‐CII, a two‐factor model was tested for self‐care maintenance (factor 1: health‐promoting behaviours; factor 2: illness‐related behaviours), a single‐factor model was tested for self‐care monitoring, and a two‐factor model was tested for self‐care management (factor 1: autonomous behaviours; factor 2: consulting behaviours) (De Maria et al. 2021; Riegel et al. 2018). Specifically, for self‐care management, we tested two models, one including item #16 in the ‘autonomous behaviours’ factor (De Maria et al. 2021) and another one with the same item included in the ‘consulting behaviours’ factor (Riegel et al. 2018). The following fit indices were considered to evaluate the model fit (Kline 2023): comparative fit index (CFI) and the Tucker‐Lewis index (TLI), with values of equal to or greater than 0.90 indicating good fit; standardised root mean square residual (SRMR), whit values of equal to or less than 0.08 indicating a good fit; root mean square error of approximation (RMSEA), whit values of less than 0.08 indicating good fit and values higher than 0.10 a poor fit; and chi‐square statistics. Since the *p*‐value of the *χ* ^2^ statistic tends to be significant when the sample size is large, it was not used to evaluate the model fit. Moreover, *p*‐value is not available in R for ULS estimator.

### 3.7 Construct Validity

The construct validity was tested by comparing the self‐care maintenance, monitoring, management, and SE‐SC instruments with the global health status dimension of the EORTC QLQ‐C30 (Scott et al. 2008) via Pearson's correlation coefficient ‘*r*’. Correlations ranging from 0.10 to 0.29 were classified as weak, those from 0.30 to 0.49 as moderate, and values equal to or greater than 0.50 were considered strong.

### 3.8 Reliability

The reliability of the unidimensional scale (self‐care monitoring and SE‐SC) was analysed with Cronbach's alpha, while the reliability of multidimensional scales (self‐care maintenance and self‐care management) was assessed with McDonald's Omega. A coefficient of 0.70 or higher indicates a satisfactory level of internal consistency, while a coefficient of 0.80 or higher suggests a high level of internal consistency. The item‐total correlation coefficients were calculated to test discrimination of items; values equal to or greater than 0.3 were considered to be acceptable. Test–retest reliability was assessed in a sample of 264 patients after 3 months with the Intraclass Correlation Coefficient (ICC). The ICC Values between 0.75 and 0.9 indicate good reliability, while values > 0.90 indicate excellent reliability.

## 4 Results

### 4.1 Clinical and Sociodemographic Characteristics of the Participants

A total of 314 patients with cancer were included in the analysis (Table 1). The participants had a median age of 55.5 years (IQR 50–69). The sample was comprised of 53.82% males and 43.59% had a high school education. Most of the patients had breast (36.89%) or genitourinary (31.72%) cancer and were treated with tyrosine kinase inhibitors (46.13%). As for inclusion criteria, all participants were treated with OAA for a median of 9 months (IQR 6–12.75) and more than half of them (53.82%) had polypharmacotherapy (intended as the intake of medicines other than OAAs). The median level of self‐care maintenance was 67.19 (IQR 50–75), self‐care monitoring was 90 (IQR 55–100), self‐care management was 70 (IQR 27.50–80) and self‐efficacy was 91.25 (IQR 60–100) (Table 1).

**TABLE 1:** Sociodemographic characteristics (*n* = 314).

	Median (IQR)	*N* (%)
Gender		
Male		169 (53.82)
Female		145 (46.18)
Age	55.5 (50–69)	
Marital status		
Single		29 (9.29)
Married/cohabiting		235 (75.32)
Divorced/separated		13 (4.17)
Widowed		35 (11.22)
Education		
None		2 (0.64)
Primary school		39 (12.50)
Secondary school		65 (20.83)
High school		136 (43.59)
Bachelor's degree		70 (22.44)
Employment		
Employee		117 (37.62)
Freelance		56 (18.01)
Householder		35 (11.25)
Retired		93 (29.90)
Unemployed		10 (3.22)
Income		
Insufficient		36 (11.46)
Sufficient		117 (37.26)
Good		159 (50.64)
Excellent		2 (0.64)
Living alone		
No		255 (81.73)
Yes		57 (18.27)
Cancer diagnosis		
Lung		38 (12.30)
Genitourinary		97 (31.72)
Breast		114 (36.89)
Gastrointestinal		32 (10.36)
Other		28 (8.74)
Oral anticancer agent		
Cytotoxic anticancer agent		77 (24.84)
Tyrosine Kinase Inhibitor		143 (46.13)
Hormonal therapy		90 (29.03)
Months of OAA therapy	9 (6–12.75)	
Take other medicines		
No		145 (46.18)
Yes (if yes, how many)	3 (2–4)	169 (53.82)
Comorbidities	1 (0–2)	
Scale scores		
Self‐care maintenance	64.29 (50–71.43)	
Self‐care monitoring	90 (55–100)	
Self‐care management	70 (27.50–80)	
Self‐care‐Self Efficacy	91.25 (60–100)	

Descriptive statistics of each item of the SC‐CII and SC‐SE are reported in Table 2. Some items (*n* = 9 for SC‐CII; *n* = 11 for SC‐SE) did not meet the criteria for univariate normality, as the skewness and kurtosis indices were > |1|. Furthermore, Mardia's test for multivariate normality confirmed a non‐normal distribution for self‐care maintenance (Skew = 1474.45, *p* < 0.001; Kurt = 36.32, *p* < 0.001), self‐care monitoring (Skew = 950.11, *p* < 0.001; Kurt = 90.32, *p* < 0.001), self‐care management (Skew = 860.57, *p* < 0.001; Kurt = 27.14, *p* < 0.001) and self‐efficacy items (Skew = 2635.56, *p* < 0.001; Kurt = 87.62, *p* < 0.001).

**TABLE 2:** Description statistics of the items composing the Self‐care of Chronic Illness Index v.2 and Self‐Care Self‐Efficacy and their respective factor loadings (*n* = 314).

	Items	Mean	SD	Skewness	Kurtosis	Primary factors loading
Self‐Care Maintenance: how often or routinely do you do the following						
1	Make sure to get enough sleep	3.44	1.00	−0.48	0.15	0.937
2	Try to avoid getting sick (e.g., flu shot, wash your hands)	3.69	1.04	−0.60	0.16	0.913
3	Do physical activity (e.g., take a brisk walk, use the stairs)	2.79	1.06	0.29	−0.30	0.429
4	Eat a special diet	3.18	1.06	−0.55	−0.20	0.713
5	See your healthcare provider for routine health care	3.72	1.09	−0.77	0.34	0.743
6	Take prescribed medicines without missing a dose	4.14	1.08	−1.17	0.75	0.957
7	Manage stress	3.02	1.26	−0.22	−0.94	0.588
Self‐Care Monitoring: how often or routinely do you do the following						
8	Monitor your condition	4.19	1.21	−1.62	−0.01	0.939
9	Pay attention to changes in how you feel	4.01	1.30	−1.06	−0.10	0.980
10	Monitor for medication side‐effects	4.06	1.26	−1.05	−0.25	0.954
11	Monitor whether you tire more than usual doing normal activities	3.98	1.28	−0.97	−0.18	0.958
12	Monitor for symptoms	4.02	1.24	−0.97	−1.23	0.963
Self‐care management						
13	If you had symptoms in the past month, how quickly did you recognise it as a symptom of your illness	2.37	1.69	−0.15	−1.23	0.897
When you have symptoms, how likely are you to…						
14	Change what you eat or drink to make the symptom decrease or go away	2.81	1.40	0.01	−1.17	0.910
15	Change your activity level (e.g., slow down, rest)	2.90	1.41	−0.08	−1.18	0.901
16	Take a medicine to make the symptom decrease or go away?	3.54	1.63	−0.60	−1.31	0.908
17	Tell your healthcare provider about the symptom at the next office visit	3.85	1.58	−0.99	−0.71	0.990
18	Call your healthcare provider for guidance	3.61	1.73	−0.67	−1.36	0.979
19	Think of a treatment you used the last time you had symptoms. Did the treatment you used make you feel better	3.13	1.82	−0.38	−1.43	0.795
Self‐Care Self‐Efficacy: in general, how confident are you that you can…						
1	Keep your chronic disease stable and free of symptoms?	3.68	1.43	−0.79	−0.73	0.947
2	Follow the treatment plan you have been given?	3.96	1.42	−1.12	−0.21	0.965
3	Persist in following the treatment plan even when difficult?	4.00	1.41	−1.15	−0.15	0.971
4	Monitor your condition routinely?	4.04	1.42	−1.20	−0.08	0.987
5	Persist in routinely monitoring your condition even when difficult?	4.00	1.40	−1.15	−0.14	0.979
6	Recognise changes in your health if they occur?	3.95	1.42	−1.07	−0.32	0.986
7	Evaluate the importance of your symptoms?	3.94	1.41	−1.06	−0.30	0.981
8	Do something to relieve your symptoms?	3.96	1.40	−1.07	−0.30	0.980
9	Persist in finding a remedy for your symptoms even when difficult?	3.92	1.43	−1.08	−0.31	0.980
10	Evaluate how well a remedy works?	3.98	1.40	−1.18	0.01	0.977

### 4.2 Self‐Care Maintenance Scale

**FIGURE 1:**
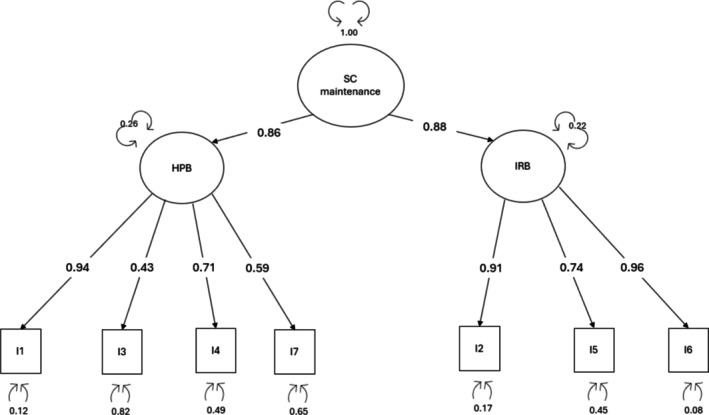
Factor loadings of the second‐order confirmatory factor analysis for self‐care maintenance scale HP, health promoting behaviours; I, item; IR, illness related behaviours; SC, self‐care.

*Dimensionality*: A two‐factor model CFA was conducted to examine the dimensionality of the self‐care maintenance scale. The ‘health‐promoting behaviours’ factor comprised items #1, #3 and #7, while the ‘illness‐related behaviours’ factor comprised items #2, #4, #5 and #6, in line with previous research (De Maria et al. 2021; Riegel et al. 2018). This model displayed an overall poor fit: *χ* ^2^ (13) = 74.553, CFI = 0.965 and TLI = 0.943, RMSEA = 0.123 (90% CI = 0.097–0.151, *p* < 0.001), and SRMR = 0.092 (Table 3). Modification indices (MI) suggested moving item #4 from the ‘illness‐related behaviours’ factor to the ‘health‐promoting behaviours’ factor. After this adjustment, the model displayed improved fit, although RMSEA remained above the ideal threshold: *χ* ^2^ (13) = 52.821, CFI = 0.977 and TLI = 0.963, RMSEA = 0.099 (90% CI = 0.072–0.127, *p* = 0.002) SRMR = 0.078. All factor loadings were significant and greater than 0.40. The correlation between factors was 0.76. Therefore, a second‐order model was tested, representing an overarching self‐care maintenance factor. This model confirmed fit indices and factor loadings > 0.40 (Figure 1).

**TABLE 3:** Confirmatory factor analysis fit indices for Self‐care of Chronic Illness Index v.2 and for Self‐care Self‐Efficacy scales.

SCALE	*χ* ^2^ (DF)	CFI	TLI	SRMR	RMSEA (90% CI)	RMSEA *p*
Self‐Care Maintenance Scale (model 1)	74.553 (13)	0.965	0.943	0.092	0.123 (0.097–0.151)	< 0.001
Self‐Care Maintenance Scale (model 2)	52.821 (13)	0.977	0.963	0.078	0.099 (0.072–0.127)	0.002
Self‐Care Monitoring Scale	0.065 (5)	1.000	1.004	0.004	0.000 (0.000–0.000	1.000
Self‐Care Management Scale (model 1)	89.360 (13)	0.977	0.963	0.101	0.137 (0.111–0.165)	< 0.001
Self‐Care Management Scale (model 2)	76.736 (13)	0.981	0.969	0.094	0.125 (0.099–0.153)	< 0.001
Self‐Care Management Scale (model 3)	19.611 (13)	0.998	0.997	0.047	0.040 (0.000–0.075)	0.636
Self‐Care Self‐Efficacy	3.253 (35)	1.000	1.003	0.014	0.000 (0.000–0.000)	1.000

Abbreviations: *χ* ^2^, Chi‐square; CFI, comparative fit index; CI, confidence interval; DF, degree of freedom; *p*, probability; RMSEA, root mean square error of approximation; SRMR, standardised root mean square residual; TLI, Tucker and Lewis index.

*Reliability*: Self‐care maintenance scale showed very good reliability at McDonald's Omega (ωt = 0.89). The item total correlation raged from 0.45 (item #7) to 0.63 (item #1) for ‘health‐promoting behaviours’ and from 0.72 (item #5) to 0.83 (item #6) for ‘illness‐related behaviours’. The ICC was 0.94 (95% CI = 0.92–0.95, *p* < 0.001), confirming supportive test–retest reliability.

### 4.3 Self‐Care Monitoring Scale

**FIGURE 2:**
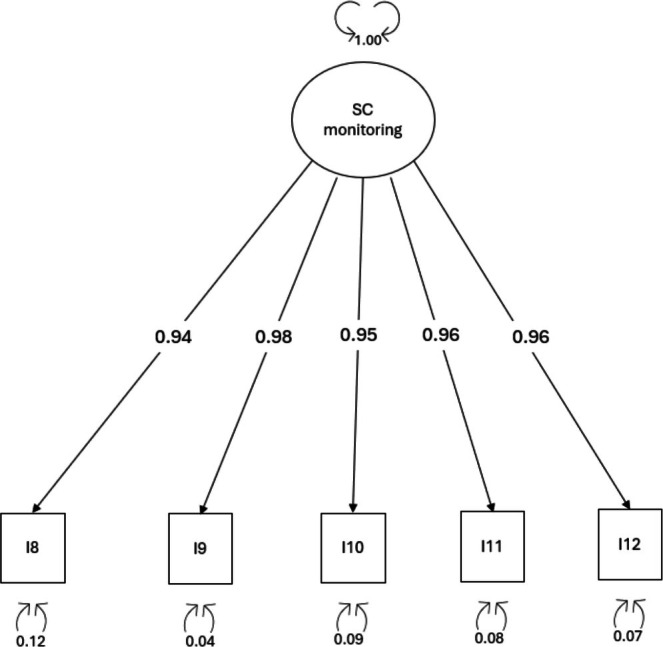
Factor loadings of the confirmatory factor analysis for self‐care monitoring scale. I, item; SC, self‐care.

*Dimensionality*: A one‐factor model CFA was tested to examine the dimensionality of the self‐care monitoring scale (Figure 2). The analysis yielded excellent fit indices: *χ* ^2^ (5) = 0.065, CFI = 1.000 and TLI = 1.004, RMSEA = 0.000 (90% CI = 0.000–0.000, *p* = 1.000) SRMR = 0.004 (Table 3). Factor loadings were all significant and > 0.90.

*Reliability*: To test the internal consistency of the scale, Cronbach's alpha coefficient was used, which yielded a value of 0.97. Item‐total correlation raged from 0.87 (item #8) to 0.94 (item #9). The test–retest reliability was confirmed with an ICC of 0.91 (95% CI = 0.89–0.93, *p* < 0.001).

### 4.4 Self‐Care Management Scale

**FIGURE 3:**
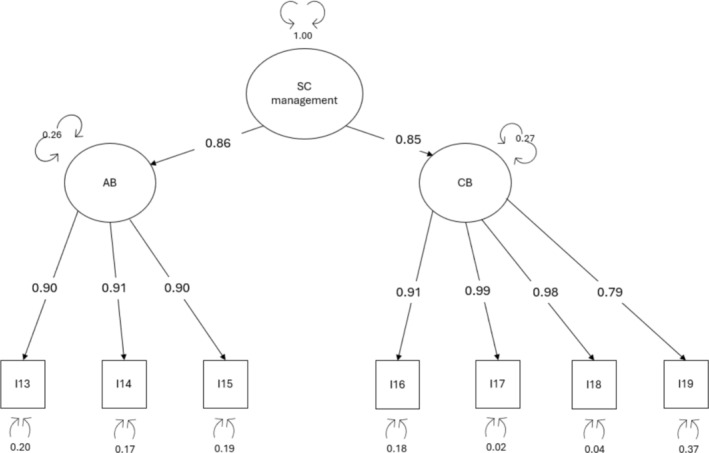
Factor loadings of the second‐order confirmatory factor analysis for self‐care management scale AB, autonomous behaviours; CB, consulting behaviours; I, item; SC, self‐care.

*Dimensionality*: Two different two‐factor models were tested. The first model (De Maria et al. 2021) included item #16 in the ‘autonomous behaviours’ factor and resulted in good fit with the exception of RMSEA: *χ* ^2^ (13) = 89.360, CFI = 0.977 and TLI = 0.963, RMSEA = 0.137 (90% CI = 0.111–0.165, *p* < 0.001) SRMR = 0.101. The second model (Riegel et al. 2018), moving item #16 to the ‘consulting behaviours’ factor, resulted in good fit again with the exception for RMSEA: *χ* ^2^ (13) = 76.736, CFI = 0.981 and TLI = 0.969, RMSEA = 0.125 (90% CI = 0.099–0.153, *p* < 0.001) SRMR = 0.094. After examining the modification indices (MI) which suggested to move item #19 from the ‘autonomous behaviours’ factor to the ‘consulting behaviours’ factor, excellent fit indices also for RMSEA were obtained: *χ* ^2^ (13) = 19.611, CFI = 0.998 and TLI = 0.997, RMSEA = 0.040 (90% CI = 0.000–0.075, *p* = 0.636) SRMR = 0.047 (Table 3). Factor loadings were all significant and greater than 0.75. The correlation between the two factors was 0.73. Thus, a second‐order model (self‐care management) was tested, yielding factor loadings > 0.75 (Figure 3).

*Reliability*: Self‐care maintenance scale showed excellent reliability (ωt = 0.96). The item‐total correlation ranged from 0.76 (item #13) to 0.86 (item #15) for ‘autonomous behaviours’ and from 0.78 (item #19) to 0.87 (item #16) for ‘consulting behaviours.’ The test–retest reliability was confirmed with an ICC of 0.85 (95% CI = 0.82–0.88, *p* < 0.001).

### 4.5 Self‐Care Self‐Efficacy Scale

**FIGURE 4:**
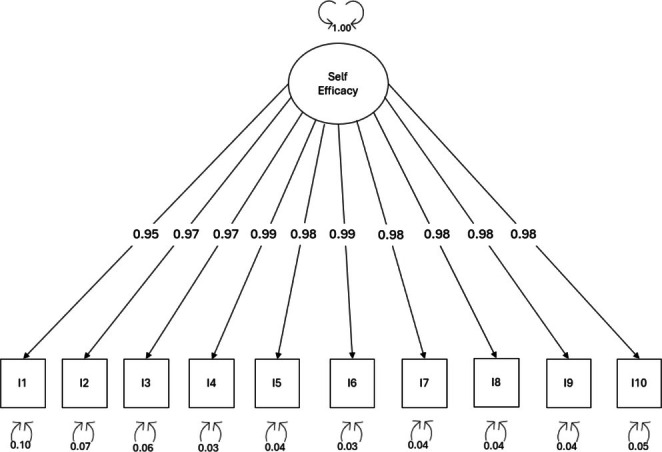
Factor loadings of the confirmatory factor analysis for Self‐Care Self‐Efficacy scale I, item.

*Dimensionality*: For the SC‐SE scale, a one‐factor CFA model was tested (Figure 4). The analysis produced excellent fit indices: *χ* ^2^ (35) = 3.253, CFI = 1.000 and TLI = 1.003, RMSEA = 0.000 (90% CI = 0.000–0.000, *p* = 1.000) SRMR = 0.014. Factor loadings were all significant and greater than 0.90.

*Reliability*: The internal consistency of the SC‐SE was tested using Cronbach's alpha coefficient, which was 0.99. Item total correlation raged from 0.91 (item #1) to 0.97 (item #6). The ICC was 0.95 (95% CI = 0.94–0.96, *p* < 0.001), confirming test–retest reliability.

### 4.6 Construct Validity

Significant positive correlations were found between the SC‐SE, each SC‐CII scale, and the global health status score using Pearson's correlations. These correlations were moderate or strong: *r* = 0.34 (*p* < 0.001) for self‐care maintenance, *r* = 0.65 (*p* < 0.001) for self‐care monitoring, *r* = 0.45 (*p* < 0.001) for self‐care management and *r* = 0.85 (*p* < 0.001) for SC‐SE.

## 5 Discussion

The purpose of this study was to rigorously evaluate the SC‐CII and SC‐SE psychometric characteristics in patients living with cancer, including dimensionality, construct validity, internal consistency, and test–retest reliability. The results, which showed good validity and reliability of the two instruments, were consistent with prior psychometric studies conducted on other populations (Arapi et al. 2023; Yu et al. 2021). To the best of our knowledge, this is the first time that these instruments were validated for patients with cancer.

### 5.1 Dimensionality and Scores

The multidimensional model of the SC‐CII was confirmed and aligned with the Middle‐Range Theory of Self‐Care of Chronic Illness (Riegel et al. 2012). Specifically, we confirmed two factors for self‐care maintenance: ‘health‐promoting behaviours’ and ‘illness‐related behaviours’. The first factor encompasses those behaviours that patients engage in to promote their overall health in their everyday life, such as getting enough sleep, doing physical activity, and managing stress (Riegel et al. 2018). These behaviours are particularly beneficial for patients with cancer dealing with chronic fatigue and reduced energy (Agbejule et al. 2022). Notably, in the domain of self‐care maintenance, the minimum average response was observed for item #3, advocating for physical activity. This phenomenon, which could be due to disease progression, adverse effects of treatment (Avancini et al. 2020), or misinformation on the topic, warrants further investigation. Since engagement in physical activity is paramount to mitigating fatigue and sustaining well‐being (Barakou et al. 2023), more significant efforts should be made to help patients with cancer engage in physical activity. Another notable result is that moving item #4 (Eat a special die) to this factor produced a better fit. This could be because, when considering chronic illnesses in general, it typically pertains to dietary restrictions or adjustments needed to manage a specific disease (e.g. low‐sodium diet for heart failure, or low‐sugar diet for diabetes). On the other hand, for patients with cancer nutritional adjustments are typically followed to maintain general wellness (e.g. increasing fresh vegetables and decreasing red and processed meat intake) sometimes without asking or informing the oncologist (Caprara et al. 2021). Therefore, this item can be thus better conceptualised as ‘health‐promoting’ rather than ‘illness‐related’ behaviours in this population.

The second factor of self‐care maintenance underscores the empowerment of daily behaviours that patients proactively engage in to manage their illness. These behaviours, such as adopting preventive measures, adhering to a specialised diet, attending healthcare appointments, and following prescribed medications, are pivotal for cancer patients, especially those undergoing OAAs. The strict adherence to prescriptions and medical appointments demonstrates the active role of patients in their health management (Di Nitto, Sollazzo, Biagioli, Pucciarelli, et al. 2022).

In the context of self‐care monitoring, we have confirmed a key factor that includes patients' behaviours aimed at monitoring their condition, how they feel, medication side‐effects, changes in energy levels during daily activities and symptoms. Many scholars have highlighted the importance of monitoring symptoms over time as a crucial part of cancer patient care, encompassing both cancer‐related symptoms and medication side effects (Di Nitto, Sollazzo, Biagioli, Torino, et al. 2022; Haase et al. 2021; Riegel et al. 2019). This monitoring process is further facilitated by the use of technology, such as eHealth applications (Shi et al. 2023).

For self‐care management, we have confirmed two factors: ‘autonomous behaviours’ and ‘consulting behaviours’. The first factor involves the actions patients choose to take based on prior experience when they recognise symptoms and need to manage them, such as changing their diet and adjusting their activity level. Patients with cancer often exhibit these innate behaviours to gain a sense of control (Adam et al. 2023). However, an intriguing pattern emerged within the ‘autonomous behaviours’ factor, wherein the mean scores were consistently < 3, whereas the mean scores of consulting behaviours were > 3. This may indicate that patients with cancer encounter difficulties in recognising symptoms related to their illness and are more likely to seek guidance and support from healthcare professionals when such symptoms occur, rather than relying on their own knowledge or past experience (Di Nitto, Sollazzo, Biagioli, Torino, et al. 2022).

These findings may have important implications for clinical practice and patient education. Identifying these overlooked behaviours could lead to targeted interventions to improve disease self‐care management capabilities, enhance self‐efficacy, and curtail unnecessary reliance on healthcare services.

The second factor encompasses behaviours healthcare providers recommend when symptoms occur, such as taking medicine, reporting the symptoms at the next visit, or calling the healthcare provider for guidance. In particular, we found that when this population reflects on the treatment they used the last time they had symptoms, this was not an ‘autonomous behaviour’ but a ‘consulting behaviour’. This may indicate that patients with cancer consider consulting with their healthcare provider as a necessity rather than a choice to manage their symptoms (Di Nitto, Sollazzo, Biagioli, Torino, et al. 2022). This result reinforces the need to promote clear communication between patients and health professionals (Mason et al. 2021), while fostering continuity of care to reduce emergency department visits (Alishahi Tabriz et al. 2023).

For the SC‐SE scale, we have confirmed one factor, including all the items in line with previous research (Fridriksdottir et al. 2023; Yu et al. 2021). This underscores the robustness of this scale in measuring self‐efficacy in patients with cancer. Therefore, the SC‐SE scale is a valid tool for researchers and clinicians to use. Enhancing patients' self‐efficacy and motivation should be the primary focus of education interventions to improve self‐care skills and coping (Been‐Dahmen et al. 2024).

### 5.2 Strengths and Limits

Our study has several strengths, notably as the first empirical examination to concurrently assess the SC‐CII and SE constructs among a cohort of patients with cancer, supported by a robust participant base. We applied sophisticated psychometric tests, adhering to stringent methodological standards, to affirm the validity and reliability of both instruments.

Nonetheless, this research presents some limitations. One such limitation is the use of a convenience sample. To address this, we made significant efforts to include a demographically diverse sample, incorporating individuals of varied genders, ages, and geographical origins across Italy's Northern, Central, and Southern regions. Despite these efforts, the generalizability of the SC‐CII and SE constructs requires further empirical tests in diverse international contexts, characterised by varying cultural values, healthcare infrastructures, and economic systems, to validate or refute our findings. Another limitation is that comprehensibility and content relevancy of all questions by patients was not assessed. However, it should be noted that this scale has been already used in several studies in cohorts of patients with chronic disease, thus items comprehensibility has been already tested. Moreover, the SC‐CII and SE have been tested only in a cohort of Italian individuals, thus future studies should confirm the validity and reliability of these scales in cohorts with different cultural background. Lastly, it is worth noting that our study focused on patients on OAA therapy, so caution should be paid when generalising to all cancer patients.

## 6 Implications

The findings of this study indicate that the two instruments are also applicable to patients with cancer. This is significant as it underscores the relevance of self‐care behaviours and self‐efficacy in self‐care theory. The possibility of having the SC‐CII and the SC‐SE scale tested for validity and reliability in the cancer population is essential for two main reasons: because cancer can be a chronic condition and both instruments have been developed for chronic conditions; and because commonly cancer patients are also affected by other chronic diseases (Fowler et al. 2020). The SC‐CII and the SC‐SE scales have been used successfully in multiple chronic conditions (De Maria et al. 2021). The SC‐CII and the SC‐SE scale can be helpful to evaluate the overall self‐care and self‐efficacy ability and to tailor nursing interventions to improve these two important variables (Sollazzo et al. 2023).

As articulated by Bandura et al. self‐efficacy pertains to the belief in one's capability to successfully manage specific tasks, circumstances, or domains of one's psychological or social functioning, which is a foundational element of self‐care (Bandura et al. 1999). Previous research looking at how Bandura's theory of self‐efficacy is applied in oncology has revealed connections between cancer prevention and self‐efficacy as well as self‐efficacy and cancer adaptability. High levels of self‐efficacy are associated with intentions to stop smoking, greater screening program participation, and the ability to cope with a cancer diagnosis. Higher levels of self‐efficacy are linked to better treatment compliance, higher levels of self‐care practices, and lower levels of psychological and physical symptoms (Lev 1997). This robust link between self‐efficacy and self‐care in oncology is a key area of knowledge for healthcare professionals, researchers, and academics in the field of oncology. In this context, nurses are in a favourable position to provide patients with feedback that could boost their sense of self‐efficacy, indirectly increasing their self‐care.

## 7 Conclusion

The Self‐Care of Chronic Illness Inventory and Self‐Care Self‐Efficacy Scale showed adequate psychometric characteristics, with supported construct validity, and confirmed factorial structures when used in patients with cancer. By using a comprehensive approach to measure self‐care behaviours in this population, health professionals can address self‐care deficits to improve by providing personalised education. These tools can also be used in future studies to measure self‐care behaviours across different diseases and cultures.

In future research, it is imperative to delve deeper into the cultural dimensions and test the scales in diverse settings that underpin the variability in meaning attribution and behaviour toward self‐care and self‐efficacy in illness management. As per previous studies on these concepts, recognising the intricate tapestry of cultural beliefs, norms, and practices is essential for a more nuanced understanding of psychometric assessments and their applicability across various populations. Such an endeavour will enhance the cultural sensitivity and validity of psychometric tools and contribute to develop more effective, culturally adapted interventions that respect and leverage the diversity of illness experiences and coping strategies. By prioritising this comprehensive approach, future research can significantly advance knowledge and practice the field, offering insights that bridge cultural divides and foster a more inclusive understanding of self‐care in cancer patients.

## Author Contributions

**Marco Di Nitto:** conceptualization, data curation, formal analysis, investigation, project administration, writing – original draft. **Angela Durante:** methodology, writing – original draft. **Francesco Torino:** conceptualization, supervision, writing – review and editing. **Tatiana Bolgeo:** writing – review and editing. **Vincenzo Damico:** writing – review and editing. **Greta Ghizzardi:** writing – review and editing. **Sipontina Rita Zerulo:** writing – review and editing. **Rosaria Alvaro:** supervision, writing – review and editing. **Ercole Vellone:** conceptualization, funding acquisition, methodology, project administration, supervision, Writing – review and editing. **Valentina Biagioli:** methodology, writing – original draft.

## Conflicts of Interest

The authors decalre no conflicts of interest.

## Supporting information


Data S1.

## Data Availability

Data can be available on reasonable request to the corresponding author.
